# Prolylcarboxypeptidase Mitigates Myocardial Ischemia/Reperfusion Injury by Stabilizing Mitophagy

**DOI:** 10.3389/fcell.2020.584933

**Published:** 2020-10-22

**Authors:** Panpan Hao, Yanping Liu, Haipeng Guo, Zhongwen Zhang, Qingjie Chen, Guoxiang Hao, Cheng Zhang, Yun Zhang

**Affiliations:** ^1^Department of Cardiology, Key Laboratory of Cardiovascular Remodeling and Function Research, Chinese Ministry of Education, Chinese National Health Commission and Chinese Academy of Medical Sciences, State and Shandong Province Joint Key Laboratory of Translational Cardiovascular Medicine, Qilu Hospital, Cheeloo College of Medicine, Shandong University, Jinan, China; ^2^Department of Radiology, Qilu Hospital, Cheeloo College of Medicine, Shandong University, Jinan, China; ^3^Shenzhen Research Institute of Shandong University, Shenzhen, China; ^4^Department of Critical Care Medicine, Qilu Hospital, Cheeloo College of Medicine, Shandong University, Jinan, China; ^5^Department of Endocrinology and Metabolism, Shandong Provincial Qianfoshan Hospital, The First Hospital Affiliated with Shandong First Medical University, Shandong University, Jinan, China; ^6^First Affiliated Hospital of Xinjiang Medical University, Ürümqi, China; ^7^Department of Clinical Pharmacy, School of Pharmaceutical Sciences, Shandong University, Jinan, China

**Keywords:** prolylcarboxypeptidase, angiotensin-(1–7), bradykinin-(1–9), ischemia, reperfusion, mitophagy

## Abstract

The role of prolylcarboxypeptidase (PRCP) in myocardial ischemia/reperfusion (I/R) injury is unclear. Herein, we aimed to evaluate the protective effect of the PRCP–angiotensin-(1–7) [Ang-(1–7)]/bradykinin-(1–9) [BK-(1–9)] axis on myocardial I/R injury and identify the mechanisms involved. Plasma PRCP level and activity, as well as Ang-(1–7) and BK-(1–9) levels, were compared in healthy subjects, patients with unstable angina, and those with ST-segment–elevated acute myocardial infarction (AMI). Thereafter, the effects of PRCP overexpression and knockdown on left ventricular function, mitophagy, and levels of Ang-(1–7) and BK-(1–9) were examined in rats during myocardial I/R. Finally, the effects of Ang-(1–7) and BK-(1–9) on I/R-induced mitophagy and the signaling pathways involved were investigated *in vitro* in rat cardiomyocytes. AMI patients showed increased plasma level and activity of PRCP and levels of Ang-(1–7) and BK-(1–9) as compared with healthy subjects and those with unstable angina. PRCP protected against myocardial I/R injury in rats by paradoxical regulation of cardiomyocyte mitophagy during the ischemia and reperfusion phases, which was mediated by downstream Ang-(1–7) and BK-(1–9). We further depicted a possible role of activation of AMPK in mitophagy induction during ischemia and activation of Akt in mitophagy inhibition during reperfusion in the beneficial effects of Ang-(1–7) and BK-(1–9). Thus, the PRCP–Ang-(1–7)/BK-(1–9) axis may protect against myocardial I/R injury by paradoxical regulation of cardiomyocyte mitophagy during ischemia and reperfusion phases.

## Introduction

A wealth of evidence suggests that the renin–angiotensin system (RAS) plays an important role in the pathophysiology of myocardial ischemia/reperfusion (I/R) injury (Mann et al., 2015; Donnarumma et al., 2016). Angiotensin II (Ang II) is upregulated after myocardial I/R and aggravates myocardial injury mediated by Ang II type 1 (AT_1_) receptor (Mann et al., 2015). Meanwhile, treatment with angiotensin-converting enzyme (ACE) inhibitors or AT_1_ receptor blockers improves I/R injury (Donnarumma et al., 2016). Recently, we found that ACE2, a zinc metalloproteinase, and its catalytic product angiotensin-(1–7) [Ang-(1–7)] provided significant cardioprotection, although the exact mechanisms are not elaborated (Dong et al., 2012; Hao P. et al., 2015). Furthermore, ACE2 overexpression inhibited hypoxia-induced collagen production by cardiofibroblasts *via* Ang-(1–7) formation (Grobe et al., 2007). The RAS may have beneficial effects against acute myocardial infarction (AMI) by maintaining the balance between the deleterious ACE–Ang II–AT_1_ receptor axis and the beneficial ACE2–Ang-(1–7)–Mas receptor axis.

The kallikrein–kinin system (KKS) is also involved in the pathophysiology of myocardial ischemic injury. The level of a core component of the KKS, bradykinin-(1–9) [BK-(1–9)], is increased in plasma of AMI patients and experimental animals, and it mitigates myocardial ischemic injury and myocyte death mediated by its B_2_ receptor (Rhaleb et al., 2011).

Autophagy is upregulated in both myocardial ischemia and reperfusion phases (Dong et al., 2019). Paradoxically, autophagy has a cardioprotective effect during ischemia but a harmful effect during reperfusion (Yao et al., 2019). Mitophagy, or autophagy of the mitochondria, facilitates the elimination of dysfunctional mitochondria by an autophagic pathway *via* selective targeting of such non-normal mitochondria (Ashrafi and Schwarz, 2013), which is important for mitochondrial quality control. Nevertheless, the precise role of mitophagy in I/R injury and the possible mechanisms behind mitophagy are unclear.

The serine protease prolylcarboxypeptidase (PRCP; also named lysosomal carboxypeptidase, angiotensinase C, or EC 3.4.16.2) is ubiquitously present in plasma and various tissues such as the heart, kidney, hypothalamus, and placenta (Chajkowski et al., 2011). Although PRCP was originally purified from lysosomes, it is constitutively expressed on the surface of the cell membrane and plays versatile roles in cell proliferation, autophagy, oxidative stress, inflammation, vascular homeostasis, and various diseases such as hypertension, obesity, diabetes, and thrombosis by metabolizing peptides including Ang II, Ang III, prekallikrein, and alpha-melanocyte-stimulating hormone (α-MSH) (Yang et al., 1968; Adams et al., 2011; Chajkowski et al., 2011; Maier et al., 2017). PRCP has protective effects on hypertension and thrombosis by activating two distinct pathways, Ang-(1–7) and BK-(1–9), and subsequently stimulating the synthesis and release of two well-known vasodilators, nitric oxide and prostaglandin (Mallela et al., 2009; Sharma, 2009; Chajkowski et al., 2011). Nevertheless, the precise roles of PRCP in myocardial I/R injury and mitophagy are unclear.

Here, we aimed to examine (1) whether PRCP protects against myocardial I/R injury by upregulating Ang-(1–7) and BK-(1–9), (2) whether mitophagy is involved in a PRCP-elicited cardioprotective effect if any, and (3) the signaling mechanisms in the PRCP- and/or mitophagy-induced myocardial response during I/R.

## Materials and Methods

### Patients

We enrolled 110 consecutive patients with ST-segment–elevated AMI caused by the culprit lesions of the left anterior descending coronary artery (LAD) only. Primary percutaneous coronary intervention (PCI) was performed within 12 h after the onset of chest pain by experienced interventionists according to the American College of Cardiology/American Heart Association guidelines for coronary angiography and PCI (Patel et al., 2017). Blood samples for measuring PRCP, Ang II, Ang-(1–7), and BK-(1–9) were collected before and 48 h after PCI. Second, we enrolled 55 age- and gender-matched participants with unstable angina, with transient ST-segment depression/elevation or T-wave inversion in the electrocardiogram, and normal cardiac troponin and CK-MB values. Third, we enrolled 110 age- and gender-matched volunteers as controls, with normal cardiac troponin and CK-MB values, negative exercise electrocardiogram, and <50% diameter stenosis by quantitative coronary angiography. The investigation conformed to the principles outlined in the Declaration of Helsinki (Br Med J 1964; ii: 177). This whole protocol was approved by the Ethics Committee of Shandong University Qilu Hospital, and all participants gave their written informed consent to participate.

### Animal Model

We obtained 120 male SPF Wistar rats (∼200 g) from Shandong University Animal Center. Rats were housed under pathogen-free conditions with a 12-h light/12-h dark cycle and given free access to water and food. After 1 week of acclimatization, rats were randomly divided into the following six groups for treatment (*n* = 20 each): sham, I/R (vehicle), adenovirus-mediated empty vector (Ad-Con), PRCP cDNA (Ad-PRCP, driven by CMV promoter), scramble shRNA (sh-Con), and PRCP shRNA (sh-PRCP, driven by U6 promoter). At the end of week 1 after gene transfer, ischemia in the left ventricular (LV) free wall was induced by ligation of the LAD for 45 min. Rats in the sham group underwent the same surgical procedure, but the suture was not tied. Thereafter, the suture was untied and rat hearts were reperfused for 4 h (Supplementary Figure 1). Ischemic repolarization changes during coronary occlusion were confirmed by electrocardiography (ST-segment elevation). Rats were euthanized and hearts were excised and stained with 2,3,5-triphenyltetrazolium chloride to delineate the extent of myocardial necrosis as a proportion of nonperfused ischemic area at risk, as described (Ma et al., 2011). The investigation conformed to the *Guide for the Care and Use of Laboratory Animals* published by the United States National Institutes of Health (NIH Publication No. 85-23, revised 1985), and the animal protocol was approved by the Institutional Animal Care and Use Committee at Shandong University Qilu Hospital.

### PRCP Protein Level and Activity

The human plasma PRCP protein level was evaluated by using the PRCP BioAssay ELISA Kit (United States Biologicals, Swampscott, MA, United States). Human plasma and rat myocardial PRCP activity were measured by the use of Ala-Pro-paranitroanilide (Lintai, Xi’an, China) as described (Chajkowski et al., 2011).

### Ang II, Ang-(1–7), and BK-(1–9) Measurement

Ang II, Ang-(1–7), and BK-(1–9) in the human and rat plasma and the rat myocardium were extracted by using C18 Sep-Pak cartridges (Waters Chromatography Division, Milford, MA, United States) and assayed by HPLC-based radioimmunoassay (Uscnlife, Wuhan, China) as described (Duncan et al., 2000).

### Echocardiography and Hemodynamic Evaluation

Before and after I/R, rats in each group underwent transthoracic echocardiographic imaging by the use of a Vevo 770 high-resolution imaging system (RMV-710B, VisualSonics, Toronto, Canada). A Millar SPR-869 microtip pressure transducer catheter (Millar Instruments, Houston, TX, United States) connected to the PowerLab system (ADInstruments, Sydney, Australia) was introduced into the left ventricle *via* the right carotid artery for measurement of heart rate, mean arterial pressure, maximal LV systolic pressure (LVSP), LV end-diastolic pressure (LVEDP), and maximal ascending and descending rate of LV pressure (±d*p*/d*t*).

### Transmission Electron Microscopy (TEM)

After hearts were excised, fresh LV tissues were quickly cut into 1-mm cubes, fixed with 2.5% glutaraldehyde, post-fixed with 1% osmium tetroxide, dehydrated through a graded ethanol series, and embedded in epoxy resin. Ultra-thin sections (90 nm) were double-stained with uranyl acetate and lead citrate, and then examined under a transmission electron microscope (model JEM-1200EX, JEOL JEM, Tokyo).

### Detection and Quantitation of Apoptosis

Paraffin-embedded sections (4-μm thick) were deparaffinized and rehydrated with serial changes of xylene and ethanol. Terminal deoxynucleotidyltransferase–mediated dUTP nick-end labeling (TUNEL) involved the use of a commercial kit (Millipore, Billerica, MA, United States). Fresh tissues were homogenized and centrifuged at 20,000 × *g* for 30 min. Caspase-3 activity was assessed in supernatants by following the proteolytic cleavage of the colorimetric substrate Ac-DEVD-ρNA (Liu et al., 2015).

### Western Blot Analysis

Heart and myocyte lysates were prepared as described (Hao P. et al., 2015). Proteins in lysates were separated on SDS-PAGE and transferred to polyvinylidene difluoride membranes (Millipore, Billerica, MA, United States), which were incubated with primary antibodies for PRCP (1:200; Santa Cruz Biotechnology, Santa Cruz, CA, United States); LC3 (1:1,000), PINK1 (1:200), Parkin (1:200), COX IV (1:1,000), p-AMPK (1:1,000), AMPKα (1:1,000), p-Akt (1:500), or Akt (1:500; all from Abcam, Cambridge, MA, United States); or β-actin (1:1,000; Cell Signaling Technology, Danvers, MA, United States), followed by appropriate horseradish peroxidase-labeled secondary antibodies. The protein level of PRCP was normalized to that of β-actin as an internal control, the levels of PINK1 and Parkin were normalized to that of COX IV, and the levels of phospho-proteins were normalized to those of total proteins.

### Statistical Analysis

SPSS v11.5 (SPSS Inc., Chicago, IL, United States) was used for statistical analysis. Continuous data were expressed as mean ± standard error (SEM) or median (interquartile range) unless otherwise stated. After testing for normality and equality of variance, intergroup differences were evaluated by one-way ANOVA, followed by Tukey-Kramer *post hoc* test and independent-samples *t* test. *p* < 0.05 was considered statistically significant.

## Results

### Circulating PRCP–Ang-(1–7)/BK-(1–9) Levels in Patients With Primary PCI

Overall, 94 patients successfully received primary PCI therapy (all drug-eluting stents), and the other 16 patients received conservative drug therapy (*n* = 11) or coronary artery bypass grafting (*n* = 5). The detailed characteristics of different groups of subjects are given in Supplementary Table 1. The plasma level and activity of PRCP and the level of BK-(1–9) were higher in AMI patients before and after PCI than healthy controls and patients with unstable angina. Plasma Ang II levels were higher in angina and AMI patients than healthy participants, and it was higher in AMI patients before PCI than angina patients. However, the Ang II level after primary PCI was similar to that in the plasma of angina patients. Additionally, plasma Ang-(1–7) levels were higher in AMI patients before and after PCI than healthy participants, and it was higher in patients after primary PCI than angina patients (Supplementary Figure 2).

### Effects of PRCP on Ang II, Ang-(1–7), and BK-(1–9) Levels in Rats

**FIGURE 1:**
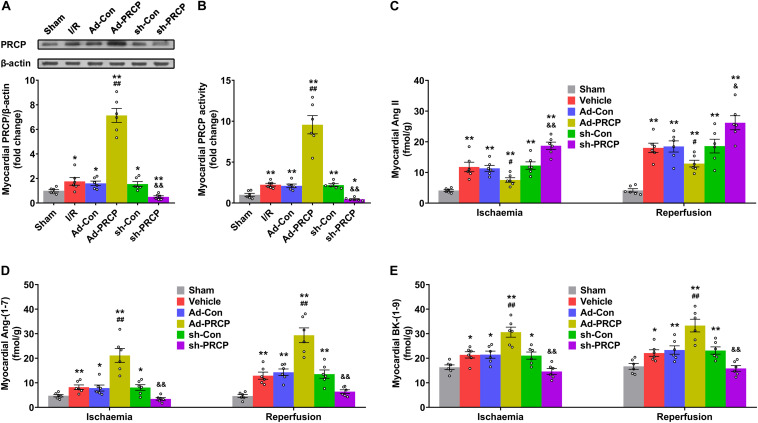
PRCP–Ang-(1–7)/BK-(1–9) changes in rat hearts after ischemia/reperfusion (I/R). **(A)** Western blot analysis of PRCP protein expression in the myocardium; **(B)** quantification of myocardial PRCP activity; **(C–E)** quantifications of myocardial Ang II **(C)**, Ang-(1–7) **(D)**, and BK-(1–9) levels **(E)** with and without overexpression (Ad) or shRNA knockdown (sh). *n* = 6 in each group; *p < 0.05 and **p < 0.01 vs. sham; ^#^*p* < 0.05 and ^##^*p* < 0.01 vs. adenovirus-mediated empty vector (Ad-Con); ^&^*p* < 0.05 and ^&⁣&^*p* < 0.01 vs. scramble shRNA (sh-Con).

Endogenous PRCP mRNA and protein expressions and enzymatic activity in the myocardium of I/R rats were higher than the sham-operated rats, which might be a compensatory response of the myocardium to I/R (Figures 1A,B and Supplementary Figure 3). PRCP expression and activity were significantly higher and lower with PRCP overexpression and knockdown, respectively, in rat hearts (Figures 1A,B and Supplementary Figure 3) as were plasma and myocardial Ang-(1–7) and BK-(1–9) levels (Figures 1D,E and Supplementary Figures 4B,C), whereas both plasma and myocardial Ang II levels were significantly lower and higher, respectively (Figure 1C and Supplementary Figure 4A). Besides, lower body weight was observed in the sh-PRCP group than the sh-Con group at the end of week 1 after gene transfer (Supplementary Figure 5).

### Effects of PRCP on Myocardial I/R Injury and LV Function

**FIGURE 2:**
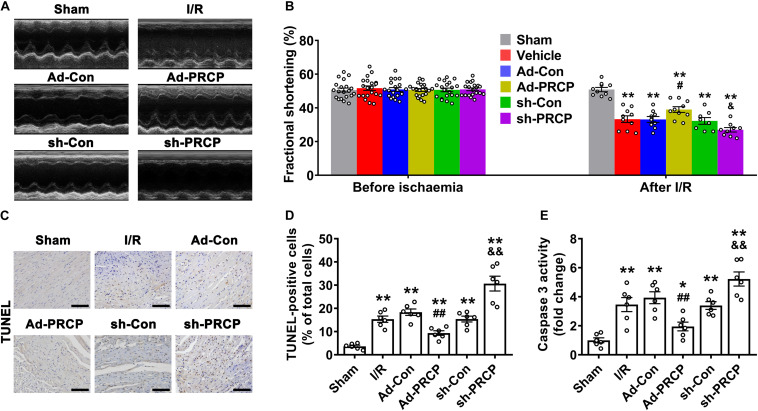
Effects of PRCP on I/R-induced left ventricular dysfunction and apoptosis. **(A)** M-mode echocardiogram showing left ventricular dimensions after myocardial I/R; **(B)** quantifications of left ventricular fractional shortening before ischemia (*n* = 20) and after reperfusion (*n* = 8∼10); **(C)** representative TUNEL staining (dark brown) for apoptosis with nuclear hematoxylin counterstaining (blue) in six groups of rats (scale bar: 100 μm); **(D)** quantification of TUNEL-positive staining (*n* = 6 in each group); **(E)** quantification of caspase-3 activity (*n* = 6 in each group). *p < 0.05 and **p < 0.01 vs. sham; ^#^*p* < 0.05 and ^##^*p* < 0.01 vs. Ad-Con; ^&^*p* < 0.05 and ^&⁣&^*p* < 0.01 vs. sh-Con.

Infarct size after I/R was significantly lower in rat hearts treated with PRCP overexpression than those with an empty vector, and it was higher in rat hearts treated with PRCP knockdown than those with scramble shRNA (Supplementary Figure 6). Echocardiography revealed significantly suppressed LV fractional shortening (FS) after I/R, which was increased with PRCP overexpression when compared with the Ad-Con group and was further decreased with PRCP knockdown in comparison with the sh-Con group (Figures 2A,B). Consistently, LV function assessed by LVSP, LVEDP, and ± d*p*/d*t* was improved with PRCP overexpression when compared with the Ad-Con group and was deteriorated with PRCP knockdown in comparison with the sh-Con group (Supplementary Figures 7A–D). The heart rate and mean arterial pressure of I/R rats were lower than those of sham-operated rats. LAD occlusion/reperfusion might decrease heart rate *via* impairing atrioventricular and/or bundle branch conduction and might induce a reduction in blood pressure secondary to decreased cardiac output and insufficient systemic vascular resistance. However, PRCP had no significant effects on heart rate and mean arterial pressure, indicating that it fails to reverse the imbalance of cardiac output and systemic vascular resistance (Supplementary Figures 7E,F).

Compared with sham rats, I/R rats showed apparent apoptosis in the border zone of hearts, and the overexpression of PRCP reduced TUNEL-positive cells and caspase-3 activity in comparison with the Ad-Con group, whereas PRCP knockdown aggravated apoptosis in comparison with the sh-Con group (Figures 2C–E).

### Effect of PRCP on Myocardial I/R-Induced Mitophagy

**FIGURE 3:**
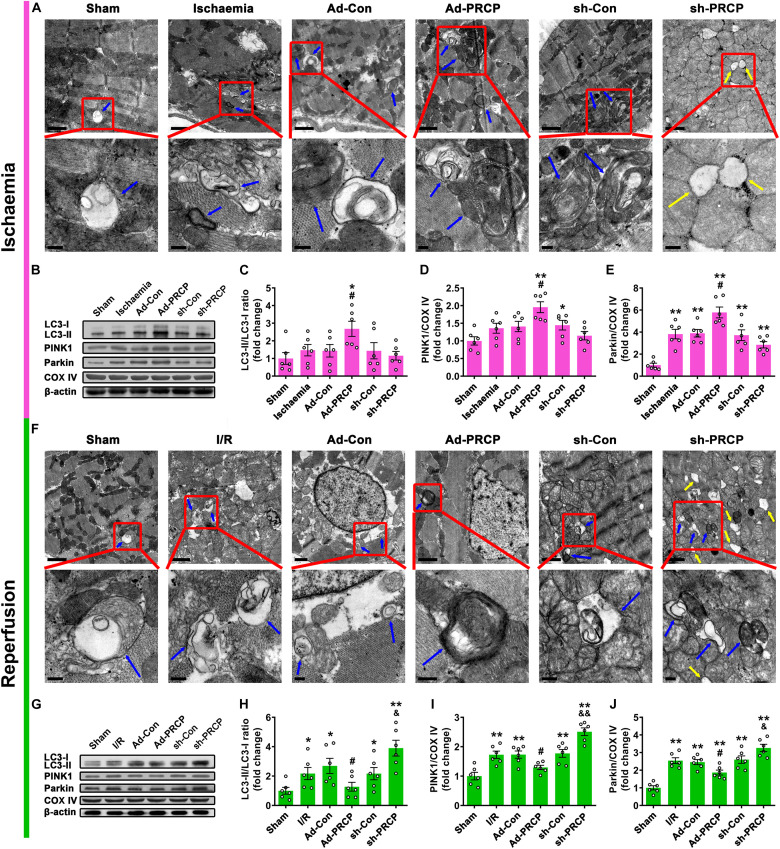
Effect of PRCP on myocardial I/R-induced mitophagy. Representative transmission electron microscopy (TEM) images of mitochondria and mitophagosomes with PRCP overexpression and knockdown in the ischemic myocardium **(A)** and reperfused myocardium **(F)** (scale bar on top panels: 1 μm; scale bar on bottom panels: 0.2 μm; blue arrows: mitophagosomes; yellow arrows: completely vacuolated mitochondria). **(B,G)** Representative Western blot analysis of LC3, PINK1, Parkin, and COX IV protein expressions in the myocardium; quantifications of LC3-II/LC3-I ratio **(C,H)** and PINK1 **(D,I)** and Parkin **(E,J)** protein expressions relative to COX IV (a mitochondrial marker). *n* = 6 in each group; *p < 0.05 and **p < 0.01 vs. sham; ^#^*p* < 0.05 vs. Ad-Con; ^&^*p* < 0.05 and ^&⁣&^*p* < 0.01 vs. sh-Con.

Transmission electron microscopy showed significant mitochondrial swelling and myofibril disarray in the I/R myocardium in comparison with the sham myocardium, and these abnormalities were improved by PRCP overexpression as compared with the Ad-Con group and aggravated by PRCP knockdown as compared with the sh-Con group (Figures 3A,F). We found concurrent increases in LC3-II/LC3-I ratio and the protein expressions of PINK1 and Parkin (mitophagy) in rat hearts under I/R in comparison with the sham hearts (Figure 3). PRCP overexpression significantly increased mitophagy under ischemia but dampened mitophagy under reperfusion as compared with the Ad-Con group, whereas PRCP knockdown significantly decreased mitophagy under ischemia but exacerbated mitophagy under reperfusion as compared with the sh-Con group.

### Effects of Ang-(1–7) and BK-(1–9) Inhibition on PRCP-Mediated Protective Effects on Cardiomyocyte Mitophagy

Both PRCP mRNA and protein expressions and enzymatic activity were significantly increased in PRCP-overexpressing rat cardiomyocytes as compared with the Ad-Con group (Supplementary Figure 8).

In line with its effect on myocardial I/R injury, PRCP overexpression effectively rescued hypoxia/reoxygenation (H/R)-induced decrease in cell viability, but this effect was reversed by inhibition of Ang-(1–7) with the Mas receptor antagonist A779 (1 μM) and AT_2_ receptor antagonist PD123319 (1 μM) or inhibition of BK-(1–9) with the B_2_ receptor antagonist HOE140 (10 μM) (Supplementary Figure 9). In line with its effect on myocardial mitophagy, PRCP overexpression significantly increased LC3-II/LC3-I ratio and protein expressions of PINK1 and Parkin under hypoxia but decreased those under reoxygenation in cultured cardiomyocytes in comparison with the Ad-Con group. The effect of PRCP on mitophagy under H/R was partially blocked by Ang-(1–7) or BK-(1–9) inhibition and completely reversed by both Ang-(1–7) and BK-(1–9) inhibition (Supplementary Figure 10).

### Effects of Ang-(1–7) and BK-(1–9) on H/R-Induced Cardiomyocyte Mitophagy

**FIGURE 4:**
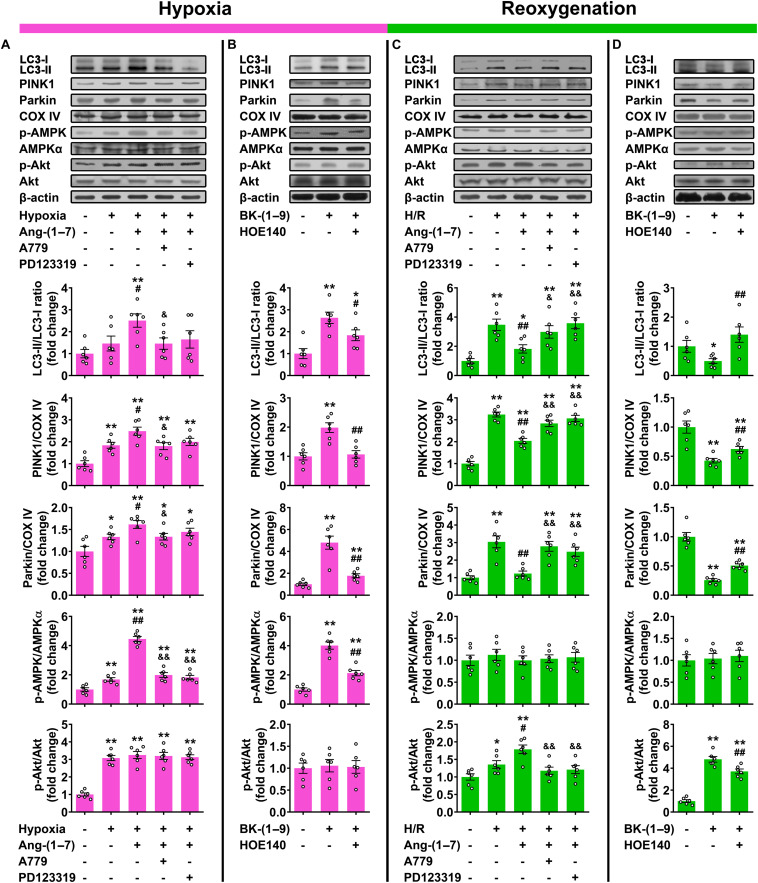
Effects of Ang-(1–7) and BK-(1–9) on cardiomyocyte mitophagy after hypoxia/reoxygenation (H/R). **(A)** Western blot analysis of LC3-II/LC3-I ratio and PINK1 and Parkin protein expressions relative to COX IV as well as AMP-dependent protein kinase (AMPK) and Akt phosphorylation in rat cardiomyocytes under normal oxygen or hypoxia and treated with vehicle, Ang-(1–7), Ang-(1–7) + A779, or Ang-(1–7) + PD123319; **(B)** under hypoxia and treated with vehicle, BK-(1–9) or BK-(1–9) + HOE140; **(C)** under normal oxygen or H/R and treated with vehicle, Ang-(1–7), Ang-(1–7) + A779 or Ang-(1–7) + PD123319; and **(D)** under H/R and treated with vehicle, BK-(1–9), or BK-(1–9) + HOE140. *n* = 6 in each group. **(A,C)** *p < 0.05 and **p < 0.01 vs. normal oxygen; ^#^*p* < 0.05 and ^##^*p* < 0.01 vs. vehicle; ^&^*p* < 0.05 and ^&⁣&^*p* < 0.01 vs. Ang-(1–7). **(B,D)** *p < 0.05 and **p < 0.01 vs. vehicle; ^#^*p* < 0.05 and ^##^*p* < 0.01 vs. BK-(1–9).

Our results showed significant concurrent increases in LC3-II/LC3-I ratio and protein expressions of PINK1 and Parkin (mitophagy) in rat cardiomyocytes under H/R as compared with those under normal oxygen (Figures 4A,C). Consistent with PRCP overexpression, treatment with 10 μM Ang-(1–7) or 10 μM BK-(1–9) significantly increased mitophagy under hypoxia (Figures 4A,B) but decreased that under reoxygenation as compared with vehicle treatment (Figures 4C,D). The mitophagy-regulating effect of Ang-(1–7) in rat cardiomyocytes was blocked by co-administration of A779 or PD123319 (Figures 4A,C) and the effect of BK-(1–9) was inhibited by co-treatment with HOE140 (Figures 4B,D).

### Mechanisms Underlying the Effects of Ang-(1–7) and BK-(1–9) on Cardiomyocyte Mitophagy

Our data revealed that hypoxia markedly increased both AMPK and Akt phosphorylation in rat cardiomyocytes as compared with those under normal oxygen (Figure 4A), and the upregulation of AMPK phosphorylation was markedly augmented by Ang-(1–7) or BK-(1–9) treatment (Figures 4A,B). The effect of Ang-(1–7) on AMPK activation under hypoxia was blocked by co-administration with A779 or PD123319, and similarly, the effect of BK-(1–9) on AMPK activation under hypoxia was inhibited by co-treatment with HOE140. However, neither Ang-(1–7) nor BK-(1–9) affected Akt phosphorylation under hypoxia as compared with vehicle treatment (Figures 4A,B).

Additionally, during reoxygenation after hypoxia, AMPK phosphorylation was rapidly decreased to basal level and unaltered by Ang-(1–7) or BK-(1–9) in rat cardiomyocytes. However, Akt phosphorylation was markedly elevated with reoxygenation with a further increase after Ang-(1–7) or BK-(1–9) treatment in cardiomyocytes (Figures 4C,D). The effect of Ang-(1–7) on Akt activation under reoxygenation was completely reversed by co-administration with A779 or PD123319, and that of BK-(1–9) was partially blocked by co-treatment with HOE140 (Figures 4C,D).

**FIGURE 5:**
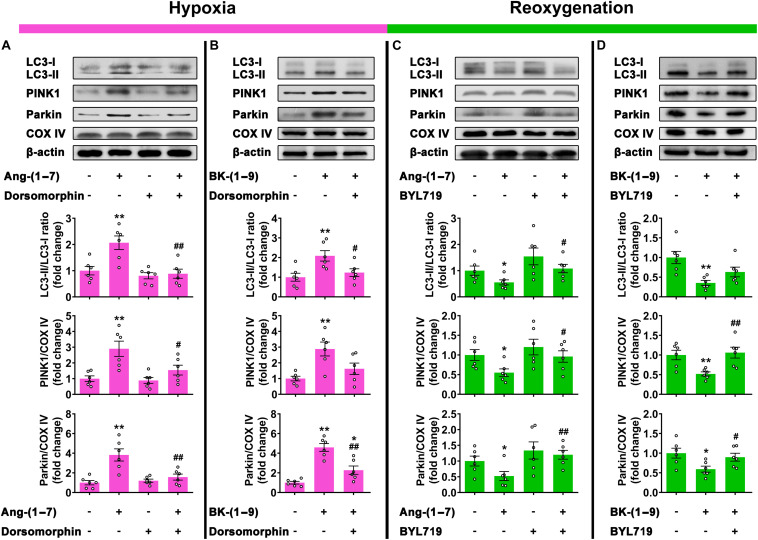
Signaling mechanisms involved in mitophagy stabilized by Ang-(1–7) and BK-(1–9). **(A)** Western blot analysis of LC3-II/LC3-I ratio and PINK1 and Parkin protein expressions relative to COX IV in rat cardiomyocytes under hypoxia and treated with vehicle, Ang-(1–7), dorsomorphin, or Ang-(1–7) + dorsomorphin; **(B)** under hypoxia and treated with vehicle, BK-(1–9), or BK-(1–9) + dorsomorphin; **(C)** under H/R and treated with vehicle, Ang-(1–7), BYL719, or Ang-(1–7) + BYL719; and **(D)** under H/R and treated with vehicle, BK-(1–9) or BK-(1–9)+ BYL719. *n* = 6 in each group; *p < 0.05 and **p < 0.01 vs. vehicle; ^#^*p* < 0.05 and ^##^*p* < 0.01 vs. Ang-(1–7) or BK-(1–9).

The effects of Ang-(1–7) and BK-(1–9) on mitophagy under hypoxia were reversed by co-administration with selective AMPK inhibitor dorsomorphin (10 μM) (Figures 5A,B), and those under reoxygenation were reversed by co-treatment with selective PI3Kα inhibitor BYL719 (25 μM) (Figures 5C,D).

## Discussion

**FIGURE 6:**
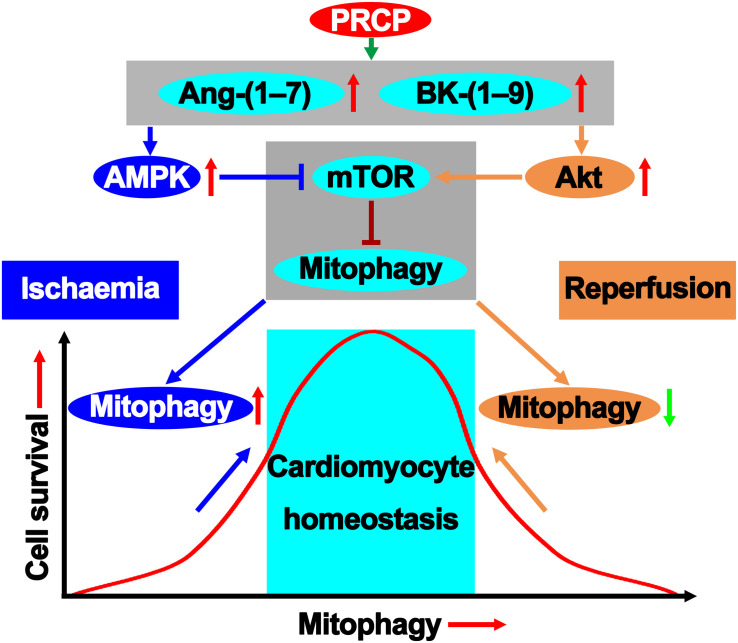
PRCP–Ang-(1–7)/BK-(1–9) modifies the mitophagy pathway in maintaining survival homeostatic response to I/R injury.

Our results demonstrated that plasma level and activity of PRCP and the levels of Ang-(1–7) and BK-(1–9) were increased in patients with ST-segment–elevated AMI and primary PCI, as compared with healthy participants and those with unstable angina, suggesting that the PRCP–Ang-(1–7)/BK-(1–9) axis might be involved in the pathogenesis of myocardial I/R injury. Moreover, PRCP protected against rat myocardial I/R injury *via* a paradoxical regulation of cardiomyocyte mitophagy during ischemia and reperfusion phases, which was mediated by downstream Ang-(1–7) and BK-(1–9) upregulation (Figure 6). Finally, we revealed a possible role for activation of AMPK in mitophagy induction during ischemia and activation of Akt in mitophagy inhibition during reperfusion in the beneficial effects of Ang-(1–7) and BK-(1–9).

To satisfy the high energy requirement, cardiomyocytes are abundant in mitochondria and, thus, vulnerable to mitochondrial damage. Mitochondrial dysfunction and cell death occur upon myocardial I/R injury, and the role of mitophagy in this process has been controversial (Ong and Gustafsson, 2012; Li et al., 2020). A major finding of the present study was that cardiomyocyte mitophagy was activated in both ischemia and reperfusion phases of AMI. Although ischemia-induced cardiomyocyte autophagy protects against myocardial ischemic injury, it may change from Doctor Jekyll to Mister Hyde in accelerating cardiomyocyte apoptosis, leading to cardiovascular dysfunction during reperfusion. Notably, Ang-(1–7) or BK-(1–9) upregulated mitophagy in the hypoxia phase but downregulated it in the reoxygenation phase as demonstrated in our study, which might be responsible for the ultimate cardioprotective effect of PRCP against I/R injury. Mechanistically, we found that the mitophagy-induction effect of Ang-(1–7) and BK-(1–9) during hypoxia was likely mediated by activation of AMPK, whereas the mitophagy-inhibition effect of Ang-(1–7) and BK-(1–9) during reoxygenation was mainly mediated by activation of Akt.

The concerted action between AMPK and Akt at the converging point of the mammalian target of rapamycin (mTOR) seems to play a pivotal role in cell survival and myocardial function (Ma et al., 2011). In the heart, both AMPK and Akt are considered key regulators of myocardial function (Liu et al., 2017). mTOR, as an important negative regulator of autophagy, is activated by Akt and inhibited by AMPK *via* phosphorylation of tuberous sclerosis complex 1/2 (TSC1/2) (Liang et al., 2018). The convergence of these two factors at the level of mTOR may be a critical avenue for the cross-talk between Akt and AMPK during pathophysiological adaptation. Our results revealed that AMPK and Akt possess different active windows during myocardial I/R. In the ischemia phase, Ang-(1–7) and BK-(1–9) activated AMPK to favor mitophagy. When AMPK is no longer active during reperfusion, Akt phosphorylation is kicked on to inhibit mitophagy. These results suggest that the dual regulatory mitophagy paradox may underscore the homeostatic machinery for Ang-(1–7)– and BK-(1–9)–elicited cardiac benefits against I/R injury. In addition to regulating mitophagy post-reperfusion, Akt activation was found to protect the heart against I/R injury by inducing mitochondrial elongation and inhibiting mitochondrial permeability transition pore (MPTP) opening (Ong et al., 2015).

Prolylcarboxypeptidase, as a member of the S28 serine peptidase family, is found on the cell surface and in lysosomes of several cell types and has both exopeptidase and endopeptidase activities, metabolizing peptides including Ang II, Ang III, prekallikrein, des-arg^9^ bradykinin, and α-MSH (Yang et al., 1968; Tan et al., 1993; Chajkowski et al., 2011). The ideal peptide substrates of PRCP contain a penultimate C-terminal proline (Yang et al., 1968), a molecular interaction attributed to the presence of two adjacent histidine residues in the active site (Soisson et al., 2010; Chajkowski et al., 2011). The PRCP crystal structure shows a previously unclassified helical SKS domain, which is unique to the S28 serine peptidase. The crystal structure also shows a classical α/β hydrolase fold. The active site includes a catalytic triad (Ser179, His455, Asp430), typical of serine proteases, between the hydrolase and SKS domains (Soisson et al., 2010). Prolylcarboxypeptidase is highly produced and ubiquitously present in plasma and versatile tissues including the kidney, heart, placenta, and hypothalamus (Chajkowski et al., 2011). PRCP expression is affected by impaired tissues within the cardiovascular system and associated with cardiovascular abnormalities and dysfunction. In a recent study, intraplaque PRCP was upregulated in unstable plaques compared to stable plaques, and PRCP transcript levels correlated positively with the reverse cholesterol transporters particularly in carotid plaque samples (Rinne et al., 2018). Circulating PRCP mainly exists in white blood cells and plasma. The correlations between serum PRCP activity and various metabolic parameters, including body mass index and subcutaneous, abdominal, and visceral adipose tissues, have been confirmed (Kehoe et al., 2018). Our data indicate that plasma PRCP level and activity are elevated in AMI but not in unstable angina. These novel findings on plasma PRCP support further investigation of its *in vivo* functions, mechanisms, and involvement as a new biomarker in AMI.

The *PRCP* gene variant promotes disease progression in hypertensive patients (Wang et al., 2006). *PRCP* depletion also induces vascular dysfunction with hypertension and faster arterial thrombosis in mice (Adams et al., 2011). Especially, global *PRCP* deficiency is associated with a moderate rise in blood pressure and alteration in the heart and kidney in mice (Maier et al., 2017). Our findings further filled in the knowledge gaps in the protective role of PRCP in myocardial injury and mitophagy and suggest that PRCP is a candidate for pharmacological intervention of myocardial injury, remodeling, and dysfunction.

The RAS and the KKS are interdependent and finely regulated. The changes in one system are obligatorily accompanied by changes in the other system. Ang-(1–7) exerts kinin-like effects and potentiates the effects of BK-(1–9), BK-(1–9) can act by modifying the actions of Ang II and Ang-(1–7), and AT_1_/AT_2_ and B_2_ receptors can form constitutive heterodimers and communicate directly with each other (Souza Dos Santos et al., 2001; Su, 2014). As shown by our results, PRCP upregulates Ang-(1–7) and BK-(1–9) and is a critical bridge for the cross-talk between the RAS and the KKS. Additionally, activation or overexpression of the AT_2_ receptor was found to increase PRCP expression and thereafter contribute to kinin release in mouse coronary artery endothelial cells, while Src homology region 2 domain-containing phosphatase 1 (SHP-1) might play a vital role in AT_2_ receptor–stimulated PRCP activation (Zhu et al., 2010, 2012).

Our study contains some limitations. First, no tissue-specific genetic knockout mice were used, and the selectivity of antagonists/inhibitors is relative. The results would be more convincing if cardiomyocyte-specific deficiency of PRCP might have been used. PRCP activators or recombinant PRCP might be more meaningful for translational medicine than gene modification and be helpful to clarify the pathophysiological changes if PRCP is activated/upregulated after ischemia or reperfusion instead of prior to ischemia. Second, we did not determine the cross-talks of PRCP with other RAS/KKS members, especially ACE, ACE2, and prolyl endopeptidase (PREP). PRCP, ACE2, and PREP convert Ang II to Ang-(1–7), and ACE metabolizes Ang-(1–7) and BK-(1–9) to inactive peptides. Whether PRCP regulates Ang-(1–7) and BK-(1–9) *via* its cross-talks with ACE, ACE2, and/or PREP is unknown. Moreover, we have found that Ang-(1–7) upregulates ACE2 and downregulates ACE in the heart of diabetic rats (Hao P. P. et al., 2015). Thus, the possibility exists that PRCP mediates its cardioprotection *via* regulating ACE and/or ACE2. *ACE*-, *ACE2*-, and *PREP*-knockout animals should be applied to investigate whether the effects of PRCP depend on these peptidases. Finally, whether other possible mechanisms besides mitophagy regulation are implicated in the cardioprotection of the PRCP–Ang-(1–7)/BK-(1–9) axis were not explored. Further studies are warranted to evaluate the role of PRCP in the cardiovascular system, in particular, the heart.

In conclusion, PRCP protects against myocardial I/R injury *via* a paradoxical regulation of cardiomyocyte mitophagy during ischemia and reperfusion phases, and its effects depend on downstream Ang-(1–7) and BK-(1–9). A possible mechanism might be mitophagy regulation in response to external stimuli, which will have fundamental importance for characterizing the cardioprotective role of the PRCP–Ang-(1–7)/BK-(1–9) axis under pathological conditions.

## Data Availability Statement

All datasets presented in this study are included in the article/Supplementary Material.

## Ethics Statement

The studies involving human participants were reviewed and approved by the Ethics Committee of Shandong University Qilu Hospital. The patients/participants provided their written informed consent to participate in this study. The animal study was reviewed and approved by the Institutional Animal Care and Use Committee at Shandong University Qilu Hospital.

## Author Contributions

PH, CZ, and YZ contributed to the study concept and design. PH, YL, and HG performed the *in vitro* work. PH, YL, ZZ, QC, and CZ performed the animal experiments. PH, YL, ZZ, QC, and GH performed the clinical studies. PH, YL, and YZ drafted the manuscript. All authors revised the manuscript and approved the final version to be published.

## Conflict of Interest

The authors declare that the research was conducted in the absence of any commercial or financial relationships that could be construed as a potential conflict of interest.
