# Supplementary material for: Prolylcarboxypeptidase Mitigates Myocardial Ischemia/Reperfusion Injury by Stabilizing Mitophagy
Source: Front Cell Dev Biol. 2020 Oct 22;8:584933. doi: 10.3389/fcell.2020.584933 (PMC7642202; doi:10.3389/fcell.2020.584933)
Supplement: Supplementary file 1 [file Data_Sheet_1.doc]

**Online data**

**Prolylcarboxypeptidase mitigates myocardial ischaemia/reperfusion injury by stabilizing mitophagy**

**Running title:** PRCP mitigates myocardial I/R injury

***By:***

*Panpan Hao^1*^, Yanping Liu^1,2^, Haipeng Guo^1,3^, Zhongwen Zhang^4^, Qingjie Chen^5^, Guoxiang Hao^6^, Cheng Zhang^1*^ and Yun Zhang^1*^*

^1^Department of Cardiology, Key Laboratory of Cardiovascular Remodeling and Function Research, Chinese Ministry of Education, Chinese National Health Commission and Chinese Academy of Medical Sciences, State & Shandong Province Joint Key Laboratory of Translational Cardiovascular Medicine, Qilu Hospital, Cheeloo College of Medicine, Shandong University, Jinan, Shandong, China

^2^Department of Radiology, Qilu Hospital, Cheeloo College of Medicine, Shandong University, Jinan, Shandong, China, and Shenzhen Research Institute of Shandong University, Shenzhen, Guangdong, China

^3^Department of Critical Care Medicine, Qilu Hospital, Cheeloo College of Medicine, Shandong University, Jinan, Shandong, China

^4^Department of Endocrinology & Metabolism, Shandong Provincial Qianfoshan Hospital, the First Hospital Affiliated with Shandong First Medical University and Shandong University, Jinan, Shandong, China

^5^First Affiliated Hospital of Xinjiang Medical University, Urumqi, Xinjiang, China

^6^Department of Clinical Pharmacy, School of Pharmaceutical Sciences, Shandong University, Jinan, Shandong, China

****Correspondence:***

Key Laboratory of Cardiovascular Remodeling and Function Research Chinese Ministry of Education and Chinese National Health Commission, Qilu Hospital, Cheeloo College of Medicine, Shandong University, Jinan, Shandong, 250012, China

**E-mail addresses:**

*Panpan Hao*

[*panda.how@sdu.edu.cn*](mailto:panda.how@sdu.edu.cn)

*Cheng Zhang*

[*zhangc_sdu@163.com*](mailto:zhangc_sdu@163.com)

*Yun Zhang*

[*zhangyun@sdu.edu.cn*](mailto:zhangyun@sdu.edu.cn)

Telephone: +86-0531-82169257

Fax: +86-0531-86169356

**Exclusion criteria of participants**

We excluded patients with previous myocardial infarction; non-ischaemic cardiomyopathy; renal or hepatic failure; malignancy, HIV, or central nervous system disorder; mechanical complication of acute myocardial infarction (AMI); cardiopulmonary resuscitation >15 min and compromised level of consciousness; cardiogenic shock; cardiac surgery planned in the next 6 months; and current participation in any study involving investigational drugs or devices.

**Gene transfection**

To increase or knockdown prolylcarboxypeptidase (PRCP) expression, 4×10^9^ plaque-forming unit (PFU)/200uL adenoviral vector carrying empty vector plasmid or PRCP cDNA (Gene ID: 293118), as well as 1×10^10^ PFU/200uL PRCP shRNA or the same volume of adenovirus vector carrying scramble shRNA (all from Genechem, Shanghai, China), was injected into the tail vein, respectively. The target sequence for PRCP shRNA was 5′-GCTACTCAGTTCATTACTTCC-3′, and the scramble control sequence was 5′-GTTCTCCGAACGTGTCACGTT-3′. The transfection efficiency was confirmed by RT-qPCR (forward primer: CTCTACGGGCTGGAGCTTTC; reverse primer: ATCCAATGAGGCCGTGGTTT).

**Echocardiography**

Before and after I/R, rats in each group underwent transthoracic echocardiographic imaging by the use of a Vevo 770 high-resolution imaging system (RMV-710B, VisualSonics, Toronto, Canada). Left ventricular end-diastolic diameter (LVEDD) and LV end-systolic diameter (LVESD) were recorded from M-mode images. LV fractional shortening (FS) was calculated as FS (%) = (LVEDD – LVESD)/LVEDD × 100%.

**Cell isolation, culture, and intervention**

Neonatal rat cardiomyocytes were isolated and cultured. First, cardiomyocytes were treated with empty adenoviral vector (Ad-Con) at a multiplicity of infection (MOI) of 100 PFU/cell, Ad-PRCP at a MOI of 100, Ad-PRCP+A779 (a selective antagonist of Mas receptor; 1μM)+PD123319 (a selective antagonist of AT_2_ receptor; 1μM), Ad-PRCP+HOE140 (a selective antagonist of bradykinin B_2_ receptor; 10μM), or Ad-PRCP+A779+PD123319+HOE140 for 24 hr at 37°C before 4-hr exposure to hypoxia (1% O_2_/5% CO_2_/94% N_2_), then with or without 4-hr reoxygenation (21% O_2_/5% CO_2_/74% N_2_). Cell viability was evaluated by the MTT method. Second, cardiomyocytes were divided into non-hypoxia group and hypoxia groups pretreated with vehicle, Ang-(1–7) (10μM), Ang-(1–7)+A779, or Ang-(1–7)+PD123319, then with or without reoxygenation. Third, cardiomyocytes were divided into vehicle, BK-(1–9) (10μM), and BK-(1–9)+HOE140 groups for treatment before hypoxia, then with or without reoxygenation. Fourth, cardiomyocytes were divided into vehicle, Ang-(1–7), dorsomorphin (a selective AMPK inhibitor; 10μM), and Ang-(1–7)+dorsomorphin groups for treatment before hypoxia without reoxygenation. Fifth, cardiomyocytes were divided into vehicle, BK-(1–9), and BK-(1–9)+dorsomorphin groups for treatment before hypoxia without reoxygenation. Sixth, cardiomyocytes were divided into vehicle, Ang-(1–7), BYL719 (a selective PI3K inhibitor; 25μM), and Ang-(1–7)+BYL719 groups for treatment before hypoxia/reoxygenation. Seventh, cardiomyocytes were divided into vehicle, BK-(1–9), and BK-(1–9)+BYL719 groups for treatment before hypoxia/reoxygenation. The scheme is shown in **Supplementary Figure 1**.

**Supplementary Table 1 Clinical characteristics of the subjects in all groups**

|  | Control  (n=110) | Unstable angina  (n=55) | STEMI | |
| --- | --- | --- | --- | --- |
|  |  |  | All  (n=110) | Primary PCI  (n=94) |
| Mean age, years | 64.19 | 64.62 | 66.95 | 66.33 |
| Men, n (%) | 82 (74.55) | 39 (70.91) | 87 (79.09) | 79 (84.04) |
| Body-mass index, kg·m^-2^ | 24.48±0.42 | 24.42±0.60 | 25.51±0.42 | 25.65±0.48 |
| Active smokers, n (%) | 38 (34.55) | 26 (47.27) | 54 (49.09) * | 43 (45.74) |
| Past medical history, n (%) |  |  |  |  |
| Diabetes | 14 (12.73) | 11 (20.00) | 26 (23.64)* | 21 (22.34) |
| Hypertension | 37 (33.64) | 28 (50.91)* | 53 (48.18)* | 46 (48.94)* |
| Dyslipidemia | 43 (39.09) | 32 (58.18)* | 67 (60.91)** | 60 (63.83)*** |
| Previous medication, n (%) |  |  |  |  |
| Antiplatelet agents | 13 (11.82) | 23 (41.82)*** | 38 (34.55)*** | 32 (34.04)*** |
| Statins | 16 (14.55) | 17 (30.91)* | 29 (26.36)* | 25 (26.60)* |
| Beta blockers | 13 (11.82) | 18 (32.73)** | 27 (24.55)* | 23 (24.47)* |
| ACE inhibitors or ARBs | 17 (15.45) | 15 (27.27) | 24 (21.82) | 21 (22.34) |

*p < 0.05, **p < 0.01 and ***p < 0.001 vs. the control group.

**Supplementary Figure Legends**

**Supplementary Figure 1**. Study protocols. Two protocols were designed in this study: 1) animal study (protocol 1); and 2) *in vitro* study (protocol 2).

**Supplementary Figure 2.** Circulating PRCP–Ang-(1–7)/BK-(1–9) changes in patients with primary PCI. Quantifications of plasma prolylcarboxypeptidase (PRCP) level **(A)** and activity **(B)**, angiotensin II (Ang II) **(C)**, angiotensin-(1–7) [Ang-(1–7)] **(D)** and bradykinin-(1–9) [BK-(1–9)] levels **(E)** in healthy controls (n = 110), patients with unstable angina (n = 55), and patients with ST-segment-elevated myocardial infarction before primary percutaneous coronary intervention (PCI) (n = 110) and after primary PCI (n = 94). **p* < 0.05 and ***p* < 0.01 vs. healthy controls; ^#^*p* < 0.05 and ^##^*p* < 0.01 vs. patients with unstable angina.

**Supplementary Figure 3.** Quantitative analysis of prolylcarboxypeptidase (PRCP) mRNA expression in rat myocardium. n = 8 in each group; ***p* < 0.01 vs. sham; ^##^*p* < 0.01 vs. adenovirus-mediated empty vector (Ad-Con); ^&&^*p* < 0.01 vs. scramble shRNA (sh-Con).

**Supplementary Figure 4.** Circulating Ang II, Ang-(1–7) and BK-(1–9) changes in rats. Quantifications of plasma angiotensin II (Ang II) **(A)**, angiotensin-(1–7) [Ang-(1–7)] **(B)** and bradykinin-(1–9) [BK-(1–9)] **(C)** levels in 6 groups of rats before gene transfer, before ischaemia, after ischaemia, and after reperfusion with and without overexpression (Ad) or shRNA knockdown (sh). n = 6 in each group; **p* < 0.05 and ***p* < 0.01 vs. sham; ^#^*p* < 0.05 and ^##^*p* < 0.01 vs. Ad-Con; ^&&^*p* < 0.01 vs. sh-Con.

**Supplementary Figure 5.** Quantitative analysis of body weight of rats before and 1 week after gene transfer (measured before ischaemia). n = 20 in each group; **p* < 0.05 vs. scramble shRNA (sh-Con).

**Supplementary Figure 6.** Effect of PRCP on myocardial infarct size after I/R.  **(A)** Representative heart sections stained with 2,3,5-triphenyltetrazolium chloride; **(B)** quantification of myocardial infarct size (as proportion of area at risk [AAR]) in 6 groups of rats (n = 6 in each group). ***p* < 0.01 vs. sham; ^#^*p* < 0.05 vs. Ad-Con; ^&^*p* < 0.05 vs. sh-Con.

**Supplementary Figure 7**. Effect of PRCP on left ventricular function assessed using a Millar catheter. Quantifications of maximal left ventricular (LV) systolic pressure (LVSP) **(A)**, LV end-diastolic pressure (LVEDP) **(B)**, maximal ascending rate of the LV pressure (+dp/dt) **(C)**, maximal descending rate of the LV pressure (-dp/dt) **(D)**, heart rate (HR) **(E)**, and mean arterial pressure (MAP) **(F)** (n = 6 in each group). **p* < 0.05 and ***p* < 0.01 vs. sham; ^#^*p* < 0.05 and ^##^*p* < 0.01 vs. Ad-Con; ^&^*p* < 0.05 and ^&&^*p* < 0.01 vs. sh-Con.

**Supplementary Figure 8.** PRCP expression and activity in rat cardiomyocytes after hypoxia/reoxygenation with or without PRCP overexpression. **(A)** Quantitative analysis of PRCP mRNA expression in rat cardiomyocytes with empty adenoviral vector (Ad-Con), Ad-PRCP, Ad-PRCP plus inhibition of Ang-(1–7) with the MasR antagonist A779 and Ang II type 2 receptor antagonist PD123319 and/or inhibition of BK-(1–9) with the B_2_ receptor antagonist HOE140; **(B)** representative Western blot analysis of PRCP protein expression in rat cardiomyocytes and quantification; **(C)** quantification of PRCP activity in rat cardiomyocytes. n = 6~8 in each group; ***p* < 0.01 vs. Ad-Con.

**Supplementary Figure 9.** Cell viability of cardiomyocytes after hypoxia/reoxygenation. Cell viability by MTT assay in rat cardiomyocytes subjected to 4-hr hypoxia or 4-hr hypoxia followed by 4-hr reoxygenation with empty vector, Ad-PRCP, Ad-PRCP+A779+PD123319, Ad-PRCP+HOE140 or Ad-PRCP+A779+PD123319+HOE140. n = 6 in each group; ***p* < 0.01 vs. Ad-Con; ^#^*p* < 0.05 and ^##^*p* < 0.01 vs. Ad-PRCP.

**Supplementary Figure 10.** PRCP regulates cardiomyocyte mitophagy after hypoxia/reoxygenation depending on Ang-(1–7) and BK-(1–9). **(A–D)** Western blot analysis of LC3-II/LC3-I ratio, and PINK1 and Parkin protein expressions relative to COX IV in rat cardiomyocytes after hypoxia and treatment with empty vector, Ad-PRCP, Ad-PRCP+A779+PD123319, Ad-PRCP+HOE140 or Ad-PRCP+A779+PD123319+HOE140; and **(E–H)** after reoxygenation and treatment with empty vector, Ad-PRCP, Ad-PRCP+A779+PD123319, Ad-PRCP+HOE140 or Ad-PRCP+A779+PD123319+HOE140. n = 6 in each group; **p* < 0.05 and ***p* < 0.01 vs. empty vector; ^#^*p* < 0.05 and ^##^*p* < 0.01 vs. Ad-PRCP.
