# Supplementary figures and images for: Prolylcarboxypeptidase Mitigates Myocardial Ischemia/Reperfusion Injury by Stabilizing Mitophagy
Source: Front Cell Dev Biol. 2020 Oct 22;8:584933. doi: 10.3389/fcell.2020.584933 (PMC7642202; doi:10.3389/fcell.2020.584933)

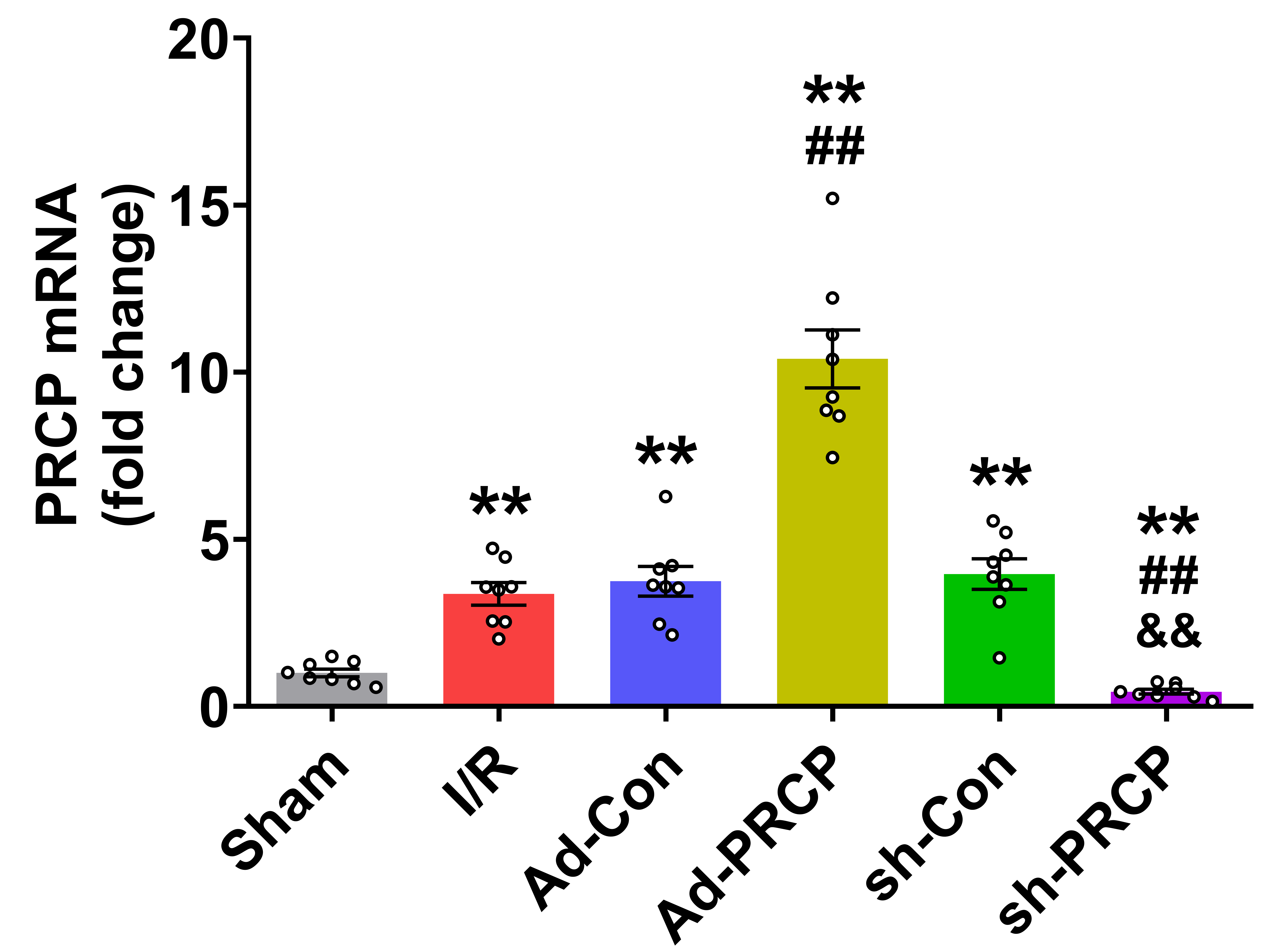

Supplement: Supplementary file 4 [file Image_3.TIF]

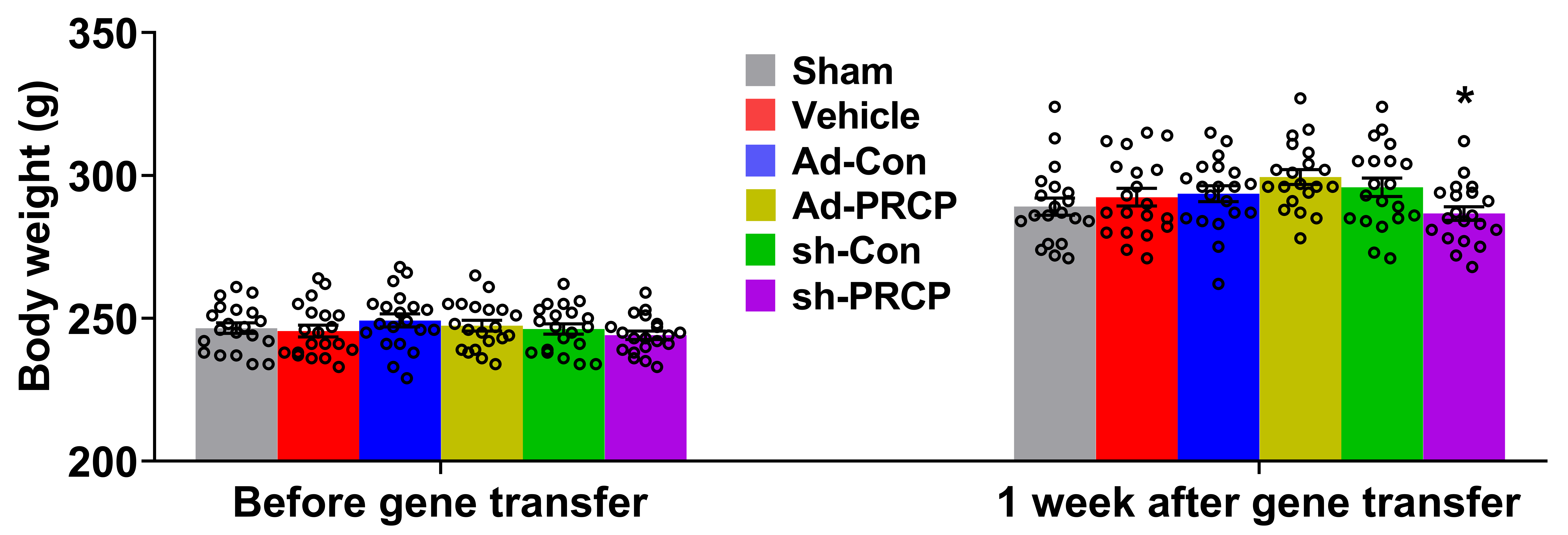

Supplement: Supplementary file 6 [file Image_5.TIF]

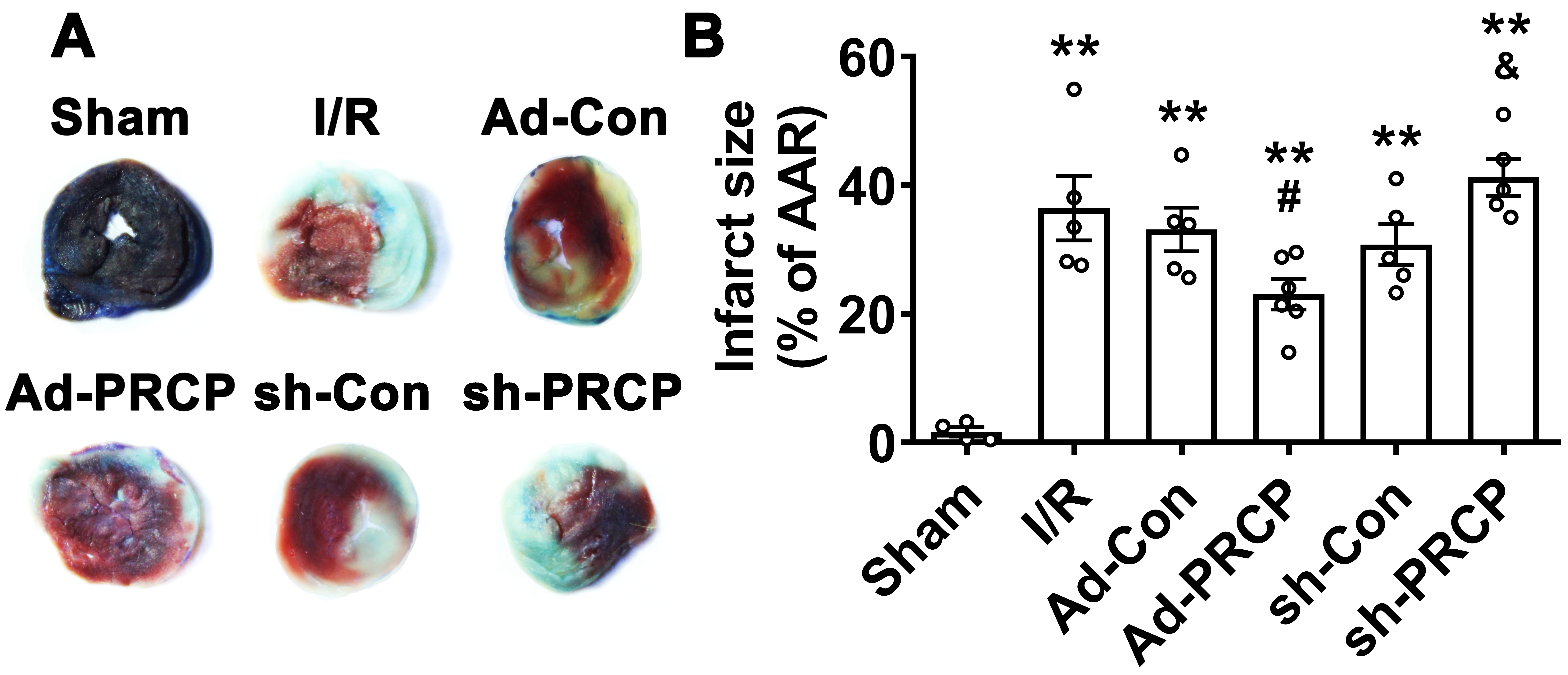

Supplement: Supplementary file 7 [file Image_6.TIF]

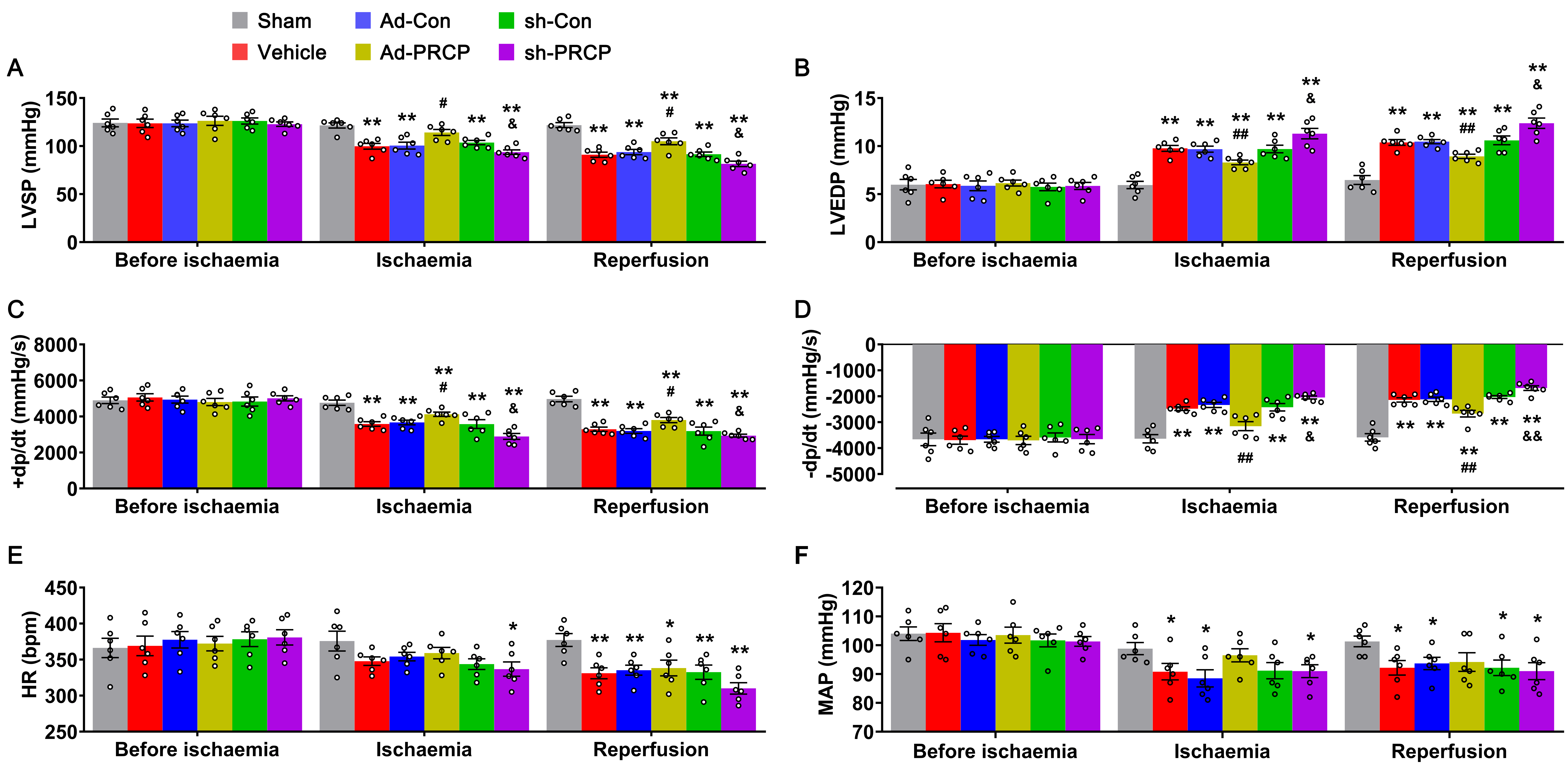

Supplement: Supplementary file 8 [file Image_7.TIF]

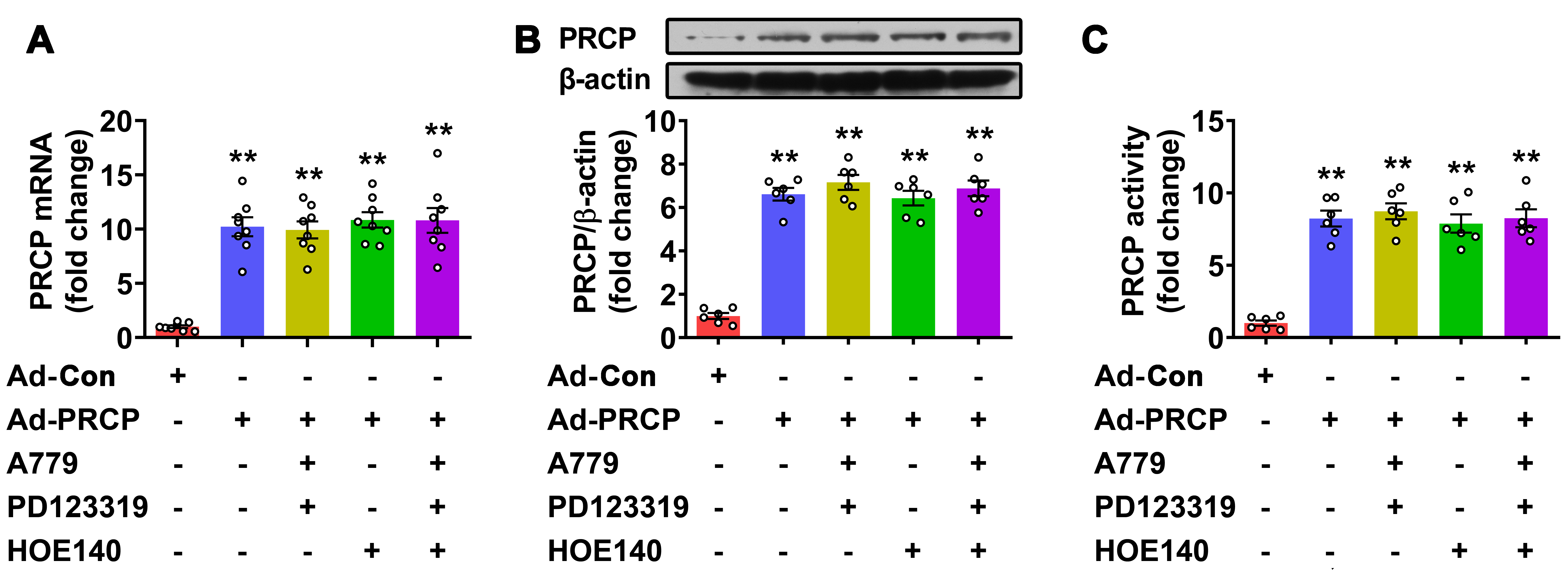

Supplement: Supplementary file 9 [file Image_8.TIF]

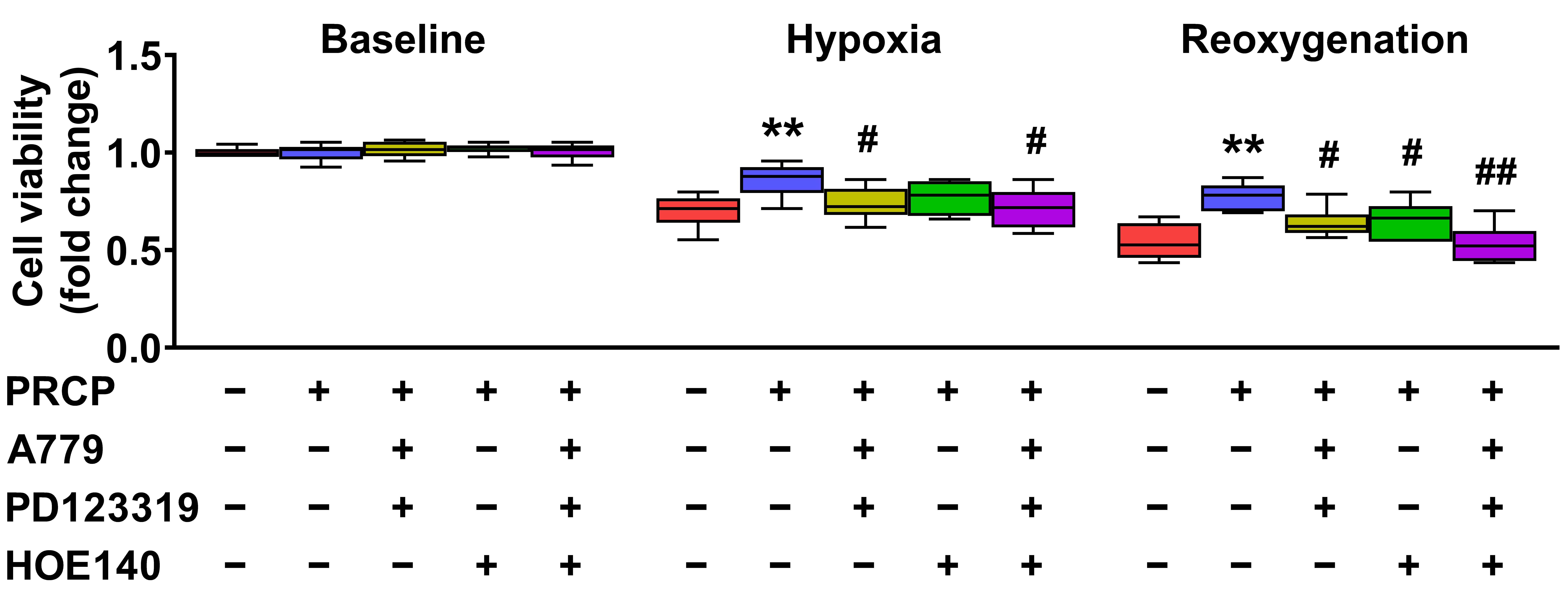

Supplement: Supplementary file 10 [file Image_9.TIF]

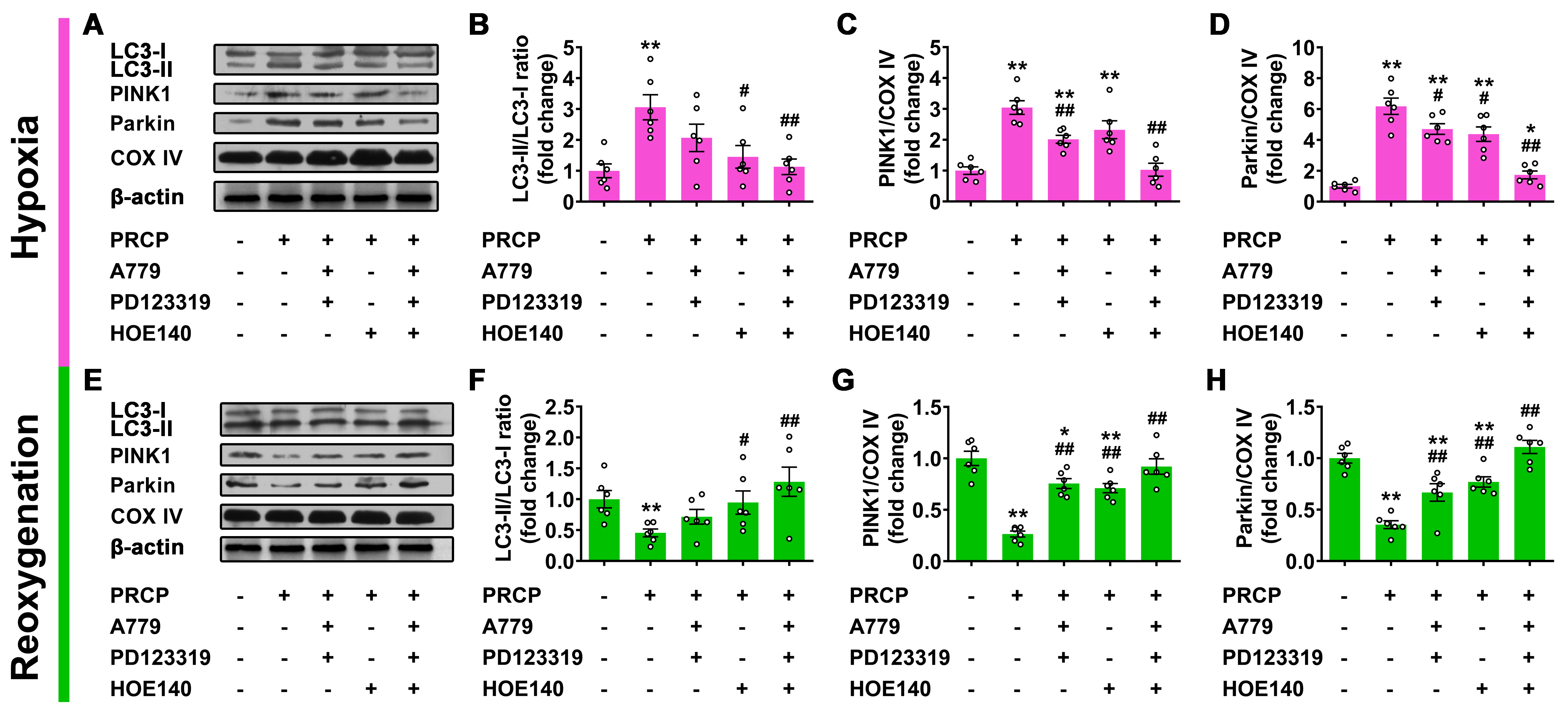

Supplement: Supplementary file 11 [file Image_10.TIF]
